# Implementation of a sexual health clinic in an oncology setting: patient and provider perspectives

**DOI:** 10.1186/s12913-024-12092-8

**Published:** 2025-01-22

**Authors:** Andrew G. Matthew, Taylor Incze, Elisa Stragapede, Steven Guirguis, Sarah E. Neil-Sztramko, Dean S. Elterman

**Affiliations:** 1 Department of Surgical Oncology, Princess Margaret Cancer Centre, University Health Network, 700 University Avenue, 6th Floor, Toronto, ON M5G 1Z6 Canada; 2 Faculty of Health Sciences, McMaster University, Hamilton, ON L8S 4K1 Canada; 3 Division of Urology, Department of Surgery, University Health Network, University of Toronto, Toronto, ON M5T 2SB Canada

**Keywords:** Sexual health, Cancer survivorship, Implementation research, CFIR, Oncosexology

## Abstract

**Background:**

Sexual dysfunction is prevalent among cancer survivors, significantly impacting patient and partner quality of life. Despite this, sexual health clinics (SHCs) remain rare in cancer centres across Canada. An innovative clinic was developed at Princess Margaret Cancer Centre in Toronto, Canada to address this significant gap in survivorship care. This study examines factors affecting the provision of sexual healthcare and the implementation of a sexual health clinic within a large urban centre.

**Methods:**

The Quality Implementation Framework was used to explicate patient and provider experience and identify barriers and facilitators to integrating sexual healthcare into routine cancer care workflows. Healthcare providers and patients representing selected cancer types (prostate, cervical, ovarian, testicular, bladder, kidney, and head and neck cancer) participated in semi-structured interviews. Interviews were transcribed and analyzed using the Framework qualitative analysis protocol.

**Results:**

The analysis identified three organizing domains and ten themes that describe the unique aspects of the sexual healthcare experience and critical factors for sexual health implementation. Both patients and providers described a lack of sexual health support in the oncology setting and emphasized the need for comprehensive and personalized care. Limitations of current care provision included mutual silence between patients and providers due to discomfort in discussing sexual issues, insufficient provider confidence in delivering optimal sexual healthcare, and constraints related to space and time. Key Factors for implementing a sexual health clinic in oncology emphasized the importance of having a dedicated clinic, flexibility in service delivery, proactive patient engagement, and ongoing staff education.

**Conclusions:**

Findings highlight significant challenges in addressing sexual health in an oncology setting, underscoring the need for specialized sexual health clinics that are integrated with, but distinct from, routine oncology care. This study further emphasizes the need for incorporating sexual healthcare in survivorship programs as well as the necessity of conducting thorough implementation research, involving multiple stakeholders, prior to launching new programs.

**Supplementary Information:**

The online version contains supplementary material available at 10.1186/s12913-024-12092-8.

## Background

Sexual dysfunction (SD) following cancer treatment significantly affects the health-related quality of life (HRQOL) of patients and their partners [1, 2]. Studies indicate that SD can result in decreased intimacy, relationship strain, and psychological distress, emphasizing its profound impact on overall well-being [3]. Despite the serious impact of SD on HRQOL, discussions about sexual health remain uncommon in oncology settings. A survey revealed that 87% of patients reported changes in sexual function or desire, yet only 27.9% had been formally asked about these concerns, highlighting a critical gap in care [4]. Furthermore, sexual health clinics (SHCs) remain rare in cancer centres across Canada, with even fewer offering empirically based comprehensive interventions [4]. This highlights the urgent need for equitable, timely, and affordable access to high-quality SD treatment for Canadian cancer patients and their partners.

The SHC at Princess Margaret Cancer Centre in Toronto, Canada, evolved from merging two established interventions: the in-person Prostate Cancer (Sexual) Rehabilitation Clinic (PCRC) [5] and the virtual Movember TrueNorth Sexual Health and Rehabilitation e-Clinic (SHAReClinic) [6] for prostate cancer patients. This innovative hybrid model of in-person and virtual care was expanded to include six additional cancer sites, with plans for further development [7]. Rooted in a biopsychosocial framework, the SHC emphasizes an interdisciplinary team and active partner involvement, offering a comprehensive approach that addresses the medical, psychological, and interpersonal aspects of sexual health. The in-person clinic, staffed by a diverse team including urologists, psychologists, nurses, and sexual health counsellors, provides tailored medical and psychosexual assessments, as well as personalized strategies to address sexual health concerns. The virtual clinic complements the in-person clinic via educational modules, symptom trackers, and goal-setting exercises. Facilitated by sexual health counsellors through chat, telephone, or videoconference, the virtual platform aims to personalize care and empower patients in self-management. Further details describing the clinic’s structure, interventions, and delivery have been previously published [7].

Despite the development of the comprehensive intervention, ensuring wide-reaching and equitable patient benefit requires effective knowledge mobilization and implementation. A fundamental link between evidence-based interventions and improvements in patient care is ensuring integration into clinical workflows. However, it is common for the adoption of research evidence in clinical practice settings to be slow and incomplete [8]. Specifically in the oncology setting, it is known that most research findings, even positive full-scale studies, do not result in practice change or take years to do so [9]. A key aspect contributing to this lack of knowledge mobilization is the complexity of transitioning from an “experimental intervention” to a “real world” clinical setting, particularly when interventions tested in a research context are not designed with real-world implementation in mind. In the context of sexual healthcare in oncology, unique complexities such as stigma, embarrassment, and limited provider training opportunities, further hinder the adaptation of interventions.

Although the current literature emphasizes the importance of addressing sexual health in oncology, it offers little consensus on how to best structure sexual health services for this population [10]. Previous sexual health clinics have demonstrated their potential to improve patient outcomes; however, they have also highlighted barriers including long wait times and resource constraints, underscoring the difficulty of integrating these interventions into existing clinical workflows [10, 11].

Recognizing these challenges, the launch of the SHC was guided by the SHC Implementation Project, utilizing the Quality Implementation Framework. This organized approach involved considerations of host sites, the creation of implementation structures, maintaining ongoing support, and refining future applications. Using a systematic qualitative investigation of interviews with key stakeholders this paper explores the barriers and facilitators of implementing an interdisciplinary SHC intervention across various sites within a high-volume cancer centre.

## Methods

### Study aim

The aim of this study was to explore the factors influencing the implementation of a SHC within a cancer centre.

### Study design

This study utilized the Quality Implementation (QI) Framework to guide knowledge mobilization and implementation strategy [12]. The QI Framework was developed though the systematic integration of 25 existing frameworks. Four pivotal steps for implementation were identified: 1) Preliminary deliberations concerning host settings, 2) Establishment of an implementation framework, 3) Maintenance of ongoing structure post-implementation, and 4) Enhancement of future applications. The QI Framework is used extensively in developing successful approaches for implementation interventions in healthcare settings. The framework typically encompasses a set of principles, processes, and steps aimed at ensuring the effective and sustainable adoption of evidence-based practices or strategies.

The methodology of this study focuses on steps 1 and 2 of the QI Framework, namely, the initial consideration regarding the host sites (e.g. testicular, ovarian), and the investigation of factors influencing the formation of a successful structured implementation plan.

### Study setting

The study setting is Princess Margaret Cancer Centre, part of the University Health Network. Princess Margaret is a scientific research centre and teaching hospital located in Toronto, Ontario Canada [13]. The Sexual Health Clinic Implementation Project was approved by UHN’s Quality Improvement board in May 2022 (ID#: 22–0393).

### Participants

Participants included both cancer survivors and healthcare providers. Eligible patient participants included cancer survivors who had completed their primary treatment from each site included in the SHC. Purposeful sampling was conducted in coordination with Princess Margaret’s Patient Engagement for Healthcare Improvement Program, a community of patients who are interested in contributing their experiences and expertise to improving and transforming healthcare. All patient partners consented to having their interviews recorded.

Eligible health care providers (HCPs) included oncologists and registered nurses from each cancer site included in the SHC. All HCPs had comprehensive knowledge of their site’s organization and patient population. They were recruited through emails sent to leadership at each site. Once contacted by the research team, they were informed about the quality improvement initiative and consented to participate and have their interviews recorded.

### Procedure

For step 1 of the QI Framework, the SHC Implementation Project team, consisting of sexual medicine specialists, oncologists, an implementation scientist, a research methodologist, a psychologist, and a patient advocate, along with their partner, participated in a one-hour unstructured group discussion. The goal of this discussion was to identify suitable host sites for inclusion in the SHC Implementation Project. Led by the principal investigator (AGM), the meeting ensured that expert opinions were gathered from all stakeholders regarding various implementation factors, such as site-based demand, practical considerations (e.g. clinic flow), and diversity in sexual health concerns, related to gender and biological sex and age.

Based on these discussions, seven specific cancer sites were chosen for inclusion: prostate, cervical, ovarian, testicular, bladder, kidney, and head & neck. Since ovarian and cervical cancers are both managed by the gynecology oncology team at Princess Margaret, these two sites were grouped for the interview process. Successful implementation across these diverse host sites was anticipated to enhance the potential for generalizing the SHC to other cancer sites.

For step 2 of the QI Framework, we interviewed key stakeholders, including patients and host site clinic personnel, regarding their preferences and approaches for the SHC integration into patient workflow (i.e. usual care). The patient and HCP semi-structured interview guides were investigator-designed based on expert opinion from the SHC Implementation Project team and examination of the scientific literature regarding knowledge translation and implementation in healthcare (see Additional file 1 and Additional file 2 for the patient and HCP interview guides, respectively). The interviews were conducted virtually and scheduled for 30 min to ensure accessibility for participants. All interviews were audio-recorded and securely stored on a password-protected server accessible only to the research team. Interviews encompassed two interconnected domains: participants first shared their insights and experiences regarding sexual healthcare in oncology, followed by a discussion of the intricacies involved in integrating an SHC into patient care procedures. This structured approach was designed to foster a conversational environment, allowing patients and providers to organically expand upon their initial responses. Ultimately, the goal of the interviews was to identify ideal methods for addressing sexual health concerns and presenting interventions according to the perspectives of both patients and healthcare professionals.

### Analysis

The QI-Framework analysis method was used to guide the qualitative analysis of the interview data [14]. This Framework has been extensively used in health services research. It is both deductive and inductive in its approach, allowing themes to be brought forward from previous literature as well as organically from the transcripts. Using a simultaneous data collection and analysis procedure, new stakeholder-participants were invited in an iterative process until data saturation was achieved. Two SHC Implementation Project team members (TI, SE) conducted and transcribed all interviews verbatim, ensuring de-identification of participant data. Four team members (AGM, TI, SE, SG) participated in the analysis of the interviews following the systematic steps as outlined in Table 1.

**Table 1:** The framework analysis method

Step	Description
Transcription	Audio recordings of the interviews are de-identified and transcribed verbatim
Familiarisation	All members of the research team read through the transcripts prior to coding
Coding	Researchers review the transcripts independently, line by line, and apply inductive and deductive codes to summarize important parts of the data
Developing a working analytical framework	All researchers involved meet to compare codes, group them as needed, and agree on a set of codes that will be applied to the remainder of the data. Discrepancies in data analysis are resolved through re-analysis of raw data (e.g. quotes) and consensus-based interpretation and abstraction
Applying the analytical framework	The analytical framework is applied to the remaining transcripts
Charting data into the framework matrix	The data is summarized by category and code and charted into a matrix for easier review
Interpreting the data	Interpretation of the codes takes place, and all members of the research team collaborate to explore characteristics and differences within the data to identify conclusions

## Results

### Data saturation

In determining knowledge translation and implementation of the SHC across the host sites, the Implementation Project team invited 19 individuals to participate in the interviews. All invitees agreed to participate resulting in a total of 19 interviews, which included six patients and 13 healthcare providers (10 oncologists and three nurses). The four analysis team members determined that continued interviewing would not achieve further unique information, and that data saturation was achieved at 19 participants.

### Identified domains and themes

After employing the QI Framework analysis method, three main domains and 10 themes were identified across both groups of participants. These are 1) Necessity for Comprehensive Sexual Healthcare in Oncology; 2) Limitations in Current Sexual Health Services in Oncology, and 3) Key Factors for Implementing Sexual Health Services in Oncology. Each of these domains had themes. Descriptions of each theme can be found below in Table 2.

**Table 2:** Domains and themes generated from qualitative analysis

Domain	Theme	Description	Quotes
Necessity for Comprehensive Sexual Healthcare in Oncology	i. Lack of Prioritization of Sexual Health	Both patients and providers described a need for sexual health support in the oncology setting	**HCP:** “I think [sexual health] is a domain that we don't cover very well in terms of our medical clinics” **Patient:** “It wasn't something that was discussed with me … I had to seek it out, so I think it would be amazing if it was sort of more normalised as part of your care”
	ii. Common Sexual Health Presentations and Standardized Care	Both patients and providers reported common sexual health concerns that were consistent across all cancer sites	**HCP:** “body image… erection issues … loss of ejaculation” **HCP:** “issues of vaginal dryness and difficulty and pain with intercourse and then stenosis sometimes if [a patient] had radiation”
	iii. Unique Sexual Health Presentations and Personalized Care	Patients and providers reported concerns that were unique to their specific cancer site	**HCP:** “a lot of our oral pharyngeal patients are caused by HPV, which is an STI, so there's a lot of stigma, anxiety, guilt, embarrassment” **HCP:** “a lot of it is psychological at that age (young adulthood) …being told that you could die and that comes with big psychological trauma and that can affect their sexual performance, particularly erections”
Limitations in Current Sexual Health Services in Oncology	iv. Mutual Silence and Avoidance	Patients and providers are unsure if sexual health is an appropriate topic to discuss. Both parties remain silent, and the topic is neglected altogether	**HCP:** “And it’s kind of taboo too, kind of a taboo topic to talk about, so having a resource that makes it not taboo where people are comfortable to talk about it is, I think, a very good thing"
	v. Fear of Providing Inadequate Care	Providers do not feel confident they have the resources to support patients in SH	**HCP:** “I don’t know if I am the right person to discuss that, if I have the training or the skill set …to address these topics appropriately”
	vi. Lack of Time	Patients and providers both report time being limited in appointments and sexual health being an item that is not prioritized	**Patient:** "I never really discussed it with her (my oncologist). I just felt like it was too long of a conversation to have…I felt like she didn't have the time… And yeah, you know, everybody's waiting. So, it's like, you don't want to take up other people's time"
Key Factors for Implementing Sexual Health Services in Oncology	vii. Unique Space and Team	Sexual health is a complex topic and cannot be comprehensively addressed in the oncology clinic setting by solely the oncology team	**HCP:** “In truth we don't really ask them about it you know, … so having a dedicated clinic …I think can have a big impact”
	viii. Engagement Flexibility	Patients have unique timelines in their cancer journeys. There is no set script for how/when sexual health should be managed	**HCP:** “Having some flexibility in the way you do things and how you deliver your intervention can be really important…So being able to deliver It virtually or with flexible hours can have an important impact” **Patients**: “I think [the SHC] should be introduced at consultation time” “[Introduce SHC] at the early follow up period “I would say [introduce SHC] after the dust has settled…after their first treatment…and they can focus on that specific issue”
	ix. Normalization Through Patient Awareness	Practitioners and patients agreed that we needed to “market” the SHC directly to patients. Patients should be active participants in their SH care	**HCP**: "Sharing stuff on the computer screens around the hospital, would be a really great way because they’re sitting in the waiting room anyways” **Patient**: "I think all of them (pamphlets, signs, posters) should be used. I mean, I think if you're sitting waiting for treatment…you have the time”
	x. Normalization Through Provider Inquiry Initially and Regularly	Multiple providers must be engaged and able to refer a patient to SHC	**HCP:** “Patients are not going to talk about it unless it's brought up so it needs to be part of their assessment, like every clinic and provider should check-in, they should ask about it and then just refer to the SHC” **HCP:** “Get the oncologists and the nurses and the radiation therapists’s knowledgeable about the sexual health clinic so we could keep on pitching it to our patients and let them know that this is the type of care that they can have, and they can get after treatment is done”

### Necessity for comprehensive sexual healthcare in oncology

Our first major domain, the *Necessity for Comprehensive Sexual Healthcare in Oncology,* encompasses the overall needs identified by patients and providers for sexual health care in oncology. There are three themes: i) Lack of Prioritization ii) Common Sexual Health Presentations and Standardized Care, and iii) Unique Sexual Health Presentations and Personalized Care.

#### Lack of prioritization of sexual health

Participants discussed the gap in care that currently exists for patients who are presenting with sexual health concerns. Providers, for instance, consistently identified that they are aware sexual health is an area seldom incorporated into the patient’s treatment journey. This acknowledgement also came with the encouragement of a structure, such as a designated clinic, to address this gap in care. Similarly, patients reported that their cancer care experience did not regularly include the provision of sexual healthcare.

#### Common sexual health presentations and standardized care

When discussing sexual health issues in oncology, participants highlighted a range of concerns that were consistent across various types of cancer. Regardless of the specific cancer diagnosis, themes such as fertility, disclosure, rebuilding intimacy with partners, and body image were frequently discussed. For instance, individuals affected by cancer and healthcare providers across different cancer types recognized the significance of visible changes to the skin and body, which could affect one's willingness to engage in sexual activity with a partner. A provider specializing in testicular cancer noted that changes in the dating process could also pose challenges for patients in disclosing the effects of cancer on their current and future sexual and reproductive health.

#### Unique sexual health presentations and personalized care

In acknowledging the typical sexual health issues faced by cancer patients, participants emphasized that sexual healthcare in oncology cannot be standardized. Patients and providers reported concerns that were unique to their specific cancer site. For instance, individuals affected by head and neck cancer highlighted unique worries about stigma and HPV, and about body image and the effect of dry mouth on kissing. Additionally, there are distinct needs related to age and gender that necessitate personalized attention. Providers were particularly attentive to the sexual health challenges younger cancer patients face, including issues related to sexual identity and self-esteem. Furthermore, participants pointed out that these distinctive sexual health challenges are exacerbated by the scarcity of resources dedicated to addressing them.

### Limitations in current sexual health services in oncology

The second domain is Limitations in Current Sexual Health Services in Oncology. This identifies the major barriers to addressing sexual health care in the oncology setting. The themes include: iv) Mutual Silence and Avoidance, v) Fear of Providing Inadequate Care, and vi) Lack of Time.

#### Mutual silence and avoidance

Often, both patients and providers are unsure if sexual health is an appropriate topic to discuss. Both parties remain silent, and the topic is neglected altogether. Some providers mentioned they were not sure if sexual health was a problem for their patients due to their advanced age; others spoke of not knowing whether to consistently follow up about sexual health concerns if initially, the patient was uninterested. Patients also reported being unsure if the oncology clinic was the appropriate place to bring concerns regarding sexual health. Overall, this theme speaks to the need for sexual health to be a more commonly addressed and acknowledged topic in oncology care.

#### Fear of providing inadequate care

This theme emerged from discussions with healthcare providers, revealing a lack of confidence in their training and resources to adequately support patients in sexual health matters. Many admitted to lacking the expertise necessary to address sexual health concerns, noting that this topic was often overlooked in their medical training. While some providers felt comfortable dealing with the physiological aspects of sexual health, they expressed discomfort with the psychosocial factors involved. As a result, many providers admitted to avoiding discussions about sexual health care due to feeling inadequately prepared to address all aspects of patients' concerns in this domain.

#### Lack of time

Providers and patients report limited time in appointments, and sexual health is an item that is not prioritized. Providers highlighted how, in a busy clinic, it is challenging to address all the issues potentially impacting a patient, while patients experienced pressure to prioritize oncology questions, believing that sexual health was not “worthy” of their oncologist’s time.

### Key factors for implementing sexual health services in oncology

The final domain identifies several distinct pathways/features needed to improve access to sexual health care in an oncology setting. Themes include: xii) Unique Space and Team, xiii) Engagement Flexibility, ix) Normalization Through Patient Awareness, and x) Normalization Through Provider Inquiry Initially and Regularly.

#### Unique space and team

The concept of separation from the oncology team and clinic was one many participants identified as possibly having a positive impact on the outcomes for patients. Patients noted that there are barriers to discussing sex and sexual difficulties with an oncologist because it is not directly related to the cancer or physical illness that a patient is presenting with. Providers emphasized this separation by acknowledging that there is a barrier to knowledge that exists among the healthcare team when it comes to sexual health. These limitations may cause more harm for patients who are seeking support related to their sexual well-being. Thus, providers noted that patients may benefit more from presenting their sexual health concerns to a trained team who can answer their questions, address their needs, and provide them with proper resources.

#### Engagement flexibility

Each patient has unique circumstances and timelines in their cancer journey. There is no set script for how and when sexual health should be managed. In this theme, providers mainly spoke to the need for the clinic to be flexible in modality. They mentioned the benefits of online care for equitable and easy access of information for their patients. Patients predominately spoke about timelines, and the need for entry into the SHC program to be flexible. The reasons for this surrounded the changing needs of patients from being directly cancer treatment-related early on to psychological and relational as time goes on. Provider’s also spoke to this. In one explanation, they described a change from patients focusing solely on getting rid of the cancer to the aftermath and ‘what now’s’ related to treatment. It was mentioned that patients from some sites may benefit from having the SHC mentioned at specific time points in the cancer journey (e.g., 18 months post-surgery for patients with testicular cancer).

#### Normalization through patient awareness

Practitioners and patients agreed that we needed to “market” the SHC directly to patients. Both parties emphasized the importance of capitalizing on waiting room times to share materials with patients and ensuring they could get information about the clinic independent of their oncology provider.

#### Normalization through provider inquiry initially and regularly

To integrate the SHC into the patient’s clinical journey through the cancer care system, participants were asked about strategies that would provide the most optimal and feasible implementation. A primary objective in these conversations was determining if there was an ideal time point in the cancer journey that would benefit patients to receive information about or be referred to the clinic. Participants identified that consistent reminders and opportunities for referral to the SHC would be not only helpful but beneficial. One participant described this method as a “continuous peppering” of mentioning the SHC. It was also suggested that engaging patients on an ongoing basis may emphasize that sexual health concerns can exist at any point in time in one’s cancer journey.

## Discussion

The implementation of a Sexual Health Clinic (SHC) at Princess Margaret Cancer Centre provided detailed insights into integrating sexual health into oncology care structured around three key domains and ten themes. These domains highlighted the necessity for comprehensive sexual healthcare, limitations in current services, and critical factors for successful implementation. Identified barriers included stigma, mutual discomfort between patients and providers, inadequate provider training, and time constraints within clinical appointments. Despite these challenges, stakeholders emphasized the importance of a dedicated sexual health service with a specialized team to provide personalized and comprehensive care. Flexible engagement strategies, proactive outreach, and the integration of sexual health discussions at multiple points throughout the cancer care continuum were identified as essential components. These findings contribute to a deeper understanding of how to address gaps in care and enhance survivorship programs by prioritizing sexual health within oncology settings.

Previous research has highlighted the importance of integrating sexual healthcare into cancer survivorship [15–18]. Our study built on this foundation by incorporating insights from implementation science literature, which identifies the necessity of gathering stakeholder perspectives before launching new programs in healthcare systems [15, 16]. By involving this crucial step, our findings offered valuable insights into the specific actions required for the successful implementation of the SHC at Princess Margaret.

The current climate for addressing sexual health in oncology settings is replete with barriers, including stigma and embarrassment, lack of medical training in sexual health, and practical limitations in current oncology treatment practices in Canada. These findings are in accordance with the previous literature [2, 3, 19, 20]. A particular challenge for providers in this study was the disparity between recognizing the need to offer sexual healthcare and feeling ill-prepared to provide comprehensive care due to time constrains and lack of relevant training. Providers noted that the training they do have in sexual health is often medicalized and prescription focused. This conceptualization of sexual health, though it can be helpful, may fail to address the underlying cognitive-emotional experience that may contribute to a patient’s sexual health concerns. For example, patients experiencing ED may be prescribed medication, but this care-response does not account for fear of resuming sexual activity, sexual performance anxiety, or anxiety regarding loss of spontaneity and natural response that may be contributing to ED. Encouraging non-medicalized conversations around sexual health may improve patient outcomes and intimate relationships. The SHC aims to address these barriers through education and knowledge translation from the sexual health team directly to HCPs and patients.

Beyond this, implementing a SHC offers benefits for both patients and providers. Patients will have direct access to a specialized team that can address their sexual health concerns and needs from diagnosis through survivorship. This will provide patients with the opportunity to discuss their stigmatized concerns in a safe space. For providers, having a designated SHC will reduce oncology clinic visit times by transitioning patients’ sexual health needs to specialized care from trained professionals. Additionally, providers will benefit from having a referral resource, which will encourage conversations on sexual health while relieving them of the sole responsibility for providing sexual health care. In this work, as well as in previous literature, primary concerns identified by patients and providers include the avoidance of sexual health conversations due to time constraints and uncertainty about the appropriateness of the topic [3, 10, 11, 21]. By implementing a SHC, it is hoped that these conversations will be streamlined to the appropriate resource, providing patients a designated time and space for these important conversations.

During interviews with participants, it became clear that there may be benefit in using multiple outreach methods, including pamphlets, posters, websites/online resources, and word-of-mouth. Incorporating multiple strategies encourages accessibility and inclusivity in reaching patients. Additionally, existing programs are often introduced at only one specific point in the patient’s journey. In contrast, patients and providers in this study advocated for continuous engagement throughout the cancer process, as sexual health concerns are not a one-size-fits-all. By offering the SHC at multiple time points, various settings, and through various avenues during the patient’s cancer journey, the SHC will be able to reach a wide range of patients experiencing sexual health challenges. Ultimately, successful implementation of the SHC can only occur if we are able to attend to the needs of patients; thus, the perspectives expressed by participants in the present work will be of utmost importance to consider and enact.

The interviews conducted with stakeholders have provided valuable insights for developing the SHC and confirmed its importance, as evidenced by the support from both patients and providers. Taking the extra step to involve stakeholders, enhances the project’s effectives and justifies its implementation. This is especially important considering that effectiveness is a key component of a successful program [22].

This study differs from previous work by capturing both patient and provider perspectives on the common and unique sexual health needs, and by emphasizing the importance of these needs as part of routine care. Successful program implementation requires a balance between effectiveness and implementation and must incorporate perspectives from various backgrounds who can provide input on the strategy’s goals [23]. The present work effectively met this requirement by conducting interviews with various stakeholders about the SHC at Princess Margaret Cancer Centre. It demonstrated that while healthcare professionals can provide valuable medical perspectives on sexual health post-cancer treatments, including patient stakeholders is essential for providing context and ensuring a comprehensive view of the implementation process.

### Strengths and limitations

This project’s strengths include its design and data analysis. In terms of design, this study is the first of its kind to incorporate the perspectives and experiences of both patient and provider stakeholders into a quality improvement project addressing the unique sexual health needs of cancer patients. Doing so allowed us to gain perspective on the experiences of patients and providers throughout the cancer journey and identified new strategies to ensure feasible and engaging implementation of the clinic. Additionally, using a Framework Analysis Method ensured a pragmatic approach to analyzing and interpreting data, which can be difficult to do in qualitative work. The results of this study emphasize the method’s benefits.

Despite the strong support for a SHC, there are limitations to this work that should be noted. First, time and availability limitations affected both researchers and stakeholders. Each interview was limited to approximately 20 to 30 min. Additionally, due to constraints in time and resources we were unable to return transcripts to participants for review, which may have provided an additional layer of validation. With more time, it is possible that other themes could have been developed beyond what was discovered. In addition, the interviews relied heavily on participants’ self-reported emotions. Discussing sexuality and sexual functioning, can be sensitive and may lead participants to feel uncomfortable discussing with strangers. In this way, it is possible that participants withheld information about their experiences or opinions so as not to disclose more than they were comfortable with. Should this be the case, our themes could be underdeveloped and possibly incomplete.

### Future directions

Results from this project will be used to shape the content and delivery of the SHC at Princess Margaret Cancer Centre. The next stage of this work will be the development of a clinic that effectively meets the needs of patients and health providers. Of course, as in all Quality Improvement work, continuous evaluation of the project will be essential to ensure success and adaptability.

## Conclusion

Overall, this study successfully identified the barriers and facilitators to implementing a SHC at Princess Margaret. The outcomes of this study provide pragmatic insight into key stakeholder enablers and barriers to many facets of integrating a SHC into patient workflow covering aspects such as accessibility, engagement, feasibility, and sustainability. Results from this project further emphasize the need for incorporating sexual healthcare into survivorship programs as well as the need for thorough implementation research, involving multiple stakeholders, prior to launching new programs.

## Supplementary Information

Additional file 1.

Additional file 2.

## Data Availability

The datasets generated and analyzed during the current study are not publicly available due to privacy and confidentiality agreements with participants but are available from the corresponding author on reasonable request.

## Abbreviations

HRQOL: Health-Related Quality of Life
SD: Sexual Dysfunction
SHC: Sexual Health Clinic
UHN: University Health Network
QI: Quality Implementation
HCPs: Healthcare Providers
