# Supplementary material for: Implementation of a sexual health clinic in an oncology setting: patient and provider perspectives
Source: BMC Health Serv Res. 2025 Jan 22;25:123. doi: 10.1186/s12913-024-12092-8 (PMC11756131; doi:10.1186/s12913-024-12092-8)
Supplement: Supplementary file 1 — Additional file 1. [file 12913_2024_12092_MOESM1_ESM.docx]

**Introductory Script and Interview Guide**

*For Interviews with Healthcare Providers*

Hello and welcome,

You are being invited to take part in an interview for a UHN Quality Improvement (QI) project on *Implementing a Sexual Health Clinic (SHC) for cancer patients at Princess Margaret Cancer Centre*. This interview is being done to gather information that will guide the implementation of a sexual health clinic that attends to the sexual health needs of cancer patients with cervical, ovarian, testicular, bladder, kidney, and head and neck cancers. The information you provide will be used to capture insight into best care and implementation practices that would allow for seamless implementation of a SHC into cancer care at PM. It will also be used to improve patient workflow while reducing provider workload.

Taking part in this interview is optional. If you decide not to participate, your employment will not be affected in any way. Information you provide will only be seen by the Project Team. Others within or outside UHN will only see a summary of the overall information collected. Your responses will not be linked to your name or personal information in any way, and will be stored separately from your personal information. It will be stored in coded form on computers accessible only to research team in a secure office for 10 years. If results of this interview are published or presented at meetings, your name and other personal identifying information will not be used, and your responses will not be linked to your name or personal information in any way.

Do you have any questions before we begin?

[No]Great. We will begin recording now.

**Background**

1. Do you feel your patients could benefit from sexual health care?
   1. If so, how?
2. Do you feel the patient population you see would benefit from a sexual health clinic?
   1. If so, how?
3. Currently, under usual care, are you aware of any sexual health care being provided to your patients?
   1. If so under what form?
4. Do you discuss sexual health with your patients?
   1. If so in what manner?
   2. Do you directly provide assistance to patients with sexual health concerns?
      1. If so, what do you provide
5. What are the most common challenges/issues your patients face with regards to their sexual health? (Inclusive of physical, emotional, relational)
6. How important do you feel sexual health care is for your patient population and why?
7. Would you be supportive of introducing a sexual health clinic to your patients and why?

**Implementation**

1. What do you believe would be the best way to integrate the SHC into patient flow?
   1. An SHC pamphlet as part of the patient education package
   2. SHC signs/posters in clinic
   3. Presented by the physician, followed by a referral
   4. Presented by clinic staff (e.g. Nurses), followed by a referral
   5. Automatic enrolment (i.e., given a pre-booked appointment) and if the patient is not interested they opt-out
   6. Self-referral
   7. Physician-referral, Staff referral and Self-referral
2. How do we best ensure clinicians/staffs are aware of the clinic and can present it to the patient if they feel the patient would benefit from the SHC?
   1. How to ensure all clinicians know about it
   2. How to ensure all patients know about it
3. How and where can SHC be added to patient flow in your clinic?
