# Supplementary material for: Implementation of a sexual health clinic in an oncology setting: patient and provider perspectives
Source: BMC Health Serv Res. 2025 Jan 22;25:123. doi: 10.1186/s12913-024-12092-8 (PMC11756131; doi:10.1186/s12913-024-12092-8)
Supplement: Supplementary file 2 — Additional file 2. [file 12913_2024_12092_MOESM2_ESM.docx]

**Introductory Script and Interview Guide**

*For Interview with Patients*

Hello and welcome,

You are being invited to take part in an interview for a UHN Quality Improvement (QI) project on *Implementing a Sexual Health Clinic (SHC) for cancer patients at Princess Margaret Cancer Centre*. This interview is being done to gather information that will guide the implementation of a sexual health clinic that attends to the sexual health needs of cancer patients with cervical, ovarian, testicular, bladder, kidney, and head and neck cancers. The information you provide will be used to capture insight into best care and implementation practices that would allow for seamless implementation of a SHC into cancer care at PM. It will also be used to improve patient workflow while reducing provider workload.

Taking part in this interview is optional. If you decide not to participate, your care will not be affected in any way. Information you provide will only be seen by the Project Team. Others within or outside UHN will only see a summary of the overall information collected. Your responses will not be linked to your name or personal information in any way, and will be stored separately from your personal health information. It will be stored in coded form on computers accessible only to research team in a secure office for 10 years. If results of this interview are published or presented at meetings, your name and other personal identifying information will not be used, and your responses will not be linked to your name or personal information in any way.

Do you have any questions before we begin?

[No]Great. We will begin recording now.

**Background**

1. We know that the diagnosis and treatment of many cancers can affect your sexual health. Since your diagnosis have you experienced any sexual health concerns?
   1. If so, what are the most common challenges/issues you’re facing in regard to your sexual health (i.e., physical, emotional, relational)?
2. Do you feel you could benefit from sexual health care?
   1. Yes or no and why?
3. Have you discussed sexual health with your oncologist or clinical team?
   1. Did you or your oncologist or clinical team initiate the discussion about your sexual health?
4. During your treatment at PM have you been made aware of any sexual health care available to you?
   1. If so, what information or services were offered?
5. If you received information or services did you find them to be beneficial?
   1. Why or why not?

**Implementation**

1. Princess Margaret is proposing the development of a sexual health clinic. Do you think this would be helpful in your cancer experience?
   1. Why or why not?
2. How would you like to be informed about the clinic?

For example:

- - 1. An SHC pamphlet as part of the patient education package
    2. SHC signs/posters in clinic
    3. Presented by the physician, followed by a referral
    4. Presented by clinic staff (e.g. Nurses), followed by a referral
    5. Automatic enrolment (i.e., given a pre-booked appointment) and if the patient is not interested, they opt-out
    6. Self-referral
    7. Physician-referral, Staff referral and Self-referral

1. At what point in your cancer care would you like to learn about it and why?
